# Different Biliary Microbial Flora Influence Type of Complications after Pancreaticoduodenectomy: A Single Center Retrospective Analysis

**DOI:** 10.3390/jcm10102180

**Published:** 2021-05-18

**Authors:** Alessandro Coppola, Vincenzo La Vaccara, Tommaso Farolfi, Michele Fiore, Chiara Cascone, Sara Ramella, Silvia Spoto, Massimo Ciccozzi, Silvia Angeletti, Roberto Coppola, Damiano Caputo

**Affiliations:** 1 Department of Surgery, Università Campus Bio-Medico di Roma, 00128 Rome, Italy; v.lavaccara@unicampus.it (V.L.V.); t.farolfi@unicampus.it (T.F.); c.cascone@unicampus.it (C.C.); r.coppola@unicampus.it (R.C.); d.caputo@unicampus.it (D.C.); 2 Radiation Oncology, Università Campus Bio-Medico di Roma, 00128 Rome, Italy; m.fiore@unicampus.it (M.F.); s.ramella@unicampus.it (S.R.); 3 Diagnostic and Therapeutic Medicine Department, Università Campus Bio-Medico di Roma, 00128 Rome, Italy; s.spoto@unicampus.it; 4 Unit of Medical Statistic and Molecular Epidemiology, Università Campus Bio-Medico di Roma, 00128 Rome, Italy; m.ciccozzi@unicampus.it; 5 Unit of Clinical Laboratory Science, Università Campus Bio-Medico di Roma, 00128 Rome, Italy; s.angeletti@unicampus.it

**Keywords:** bacterobilia, pancreaticoduodenectomy, *E. coli*, pancreatic fistula, DGE, ERCP

## Abstract

Background: Bacterobilia is associated with postoperative morbidity after pancreaticoduodenectomy (PD), mostly due to infectious complications. The aim of this study was to investigate the prevalence of bacteria species isolated from intraoperative biliary cultures, and related complications after PD. Methods: An ANOVA test was used to assess the prevalence of isolated bacterial species and postoperative complications. The odds ratio was computed to evaluate the association between biliary cultures and each complication, Endoscopic Retrograde CholangioPancreatography (ERCP) and each complication, ERCP and biliary cultures, Delayed Gastric Emptying (DGE) and Postoperative Pancreatic Fistula (POPF). Results: Positive biliary cultures were found in 162/244 (66%) PDs. Different prevalences of polymicrobial biliary culture were detected in patients with postoperative complications. In SSIs, a significant prevalence of biliary culture positive for *E. coli*, *Klebsiella pneumoniæ* and *Enterococcus fæcalis* (*p* < 0.001) was detected. Prevalences of polymicrobial biliary cultures with *Escherichia coli*, *Klebsiella pneumoniæ*, *Enterococcus fæcalis* and *Enterococcus fæcium* were significantly associated with POPF (*p* < 0.001). Biliary culture positive for *Escherichia coli*, *Enterococcus fæcalis* and *Enterococcus fæcium* showed a higher prevalence of intra-abdominal collection and DGE (*p* < 0.001). Notably, *Escherichia coli* was significantly associated with DGE as a unique complication (OR = 2.94 (1.30–6.70); *p* < 0.01). Conclusions: Specific prevalences of polymicrobial bacterobilia are associated with major complications, while monomicrobial *Escherichia coli* bacterobilia is associated with DGE as a unique complication after PD.

## 1. Introduction

Pancreatoduodenectomy (PD) represents the standard of care for periampullary malignancies. In recent years, despite technical improvements leading to a decrease in post-PD mortality, i.e., to less than 5% in high volume centers [1,2], morbidity remains high, i.e., up to 40%. Main complications include postoperative pancreatic fistula (POPF), delayed gastric emptying (DGE), hemorrhage, intra-abdominal collections, sepsis and biliary fistula [3].

The role that bacterobilia has in post-PD infectious morbidity has been widely investigated [4]. Nevertheless, bacterobilia has been associated with noninfectious postoperative complications including DGE [5] and POPF [6]. In addition, Sandini demonstrated how the presence of multidrug-resistant or extensive drug-resistant microbes are significantly associated with higher rates of POPF [7]. Moreover, Hecler reported how defined bacterial species, such as *Escherichia coli* bacterobilia, are associated with worse outcomes of POPF [8].

Even though bacterobilia is frequently observed in patients undergoing PD, and mainly in those who received preoperative biliary drainage [9], limited data are available regarding the correlation between postoperative complications after PD and different biliary bacterial species.

The aim of this study was to investigate the prevalence of different types of bacteria isolated from intraoperative biliary culture in patients with postoperative complications after PD.

## 2. Materials and Methods

**Figure 1:**
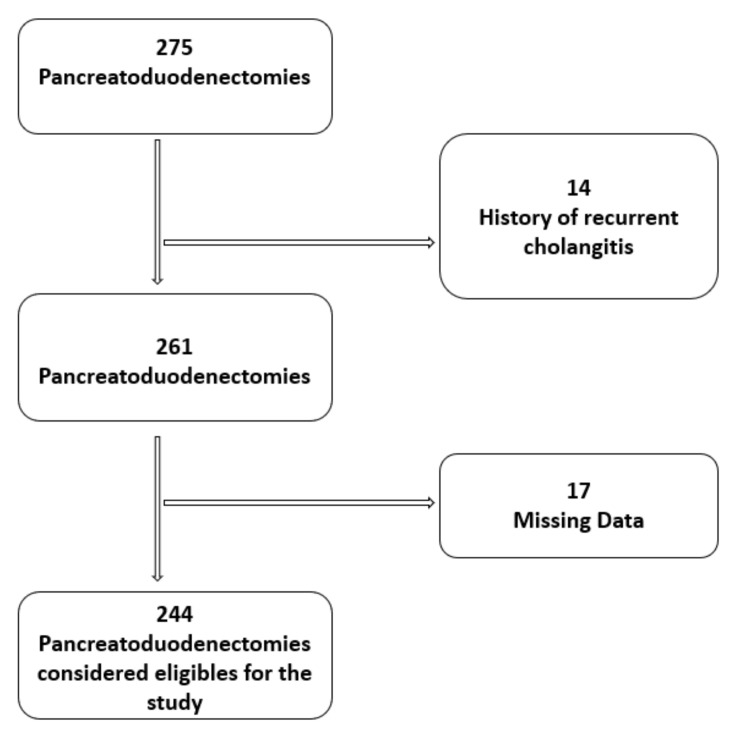
Patient selection flow-chart.

Data collected between January 2010 and December 2019, from a prospective maintained database of PDs for periampullary malignant tumors at Campus Bio-Medico University Teaching Hospital, were retrospectively analyzed (Figure 1).

The Ethical Committee of University Campus Bio-Medico di Roma approved this study (Prot.: 105.20 OSS.NOT ComEt CBM), which was carried out according to the principles of Good Clinical Practice.

Patient characteristics such as age, sex, ASA score and BMI, and data regarding preoperative management, such as preoperative biliary drainage and neoadjuvant treatment (NAT), were evaluated. Data regarding intraoperative management as a result of biliary culture, pancreatic texture, diameter of the Wirsung duct, median blood loss and operative time, as well as postoperative complications such as POPF, DGE, hemorrhage, biliary fistula, intra-abdominal collections, sepsis and surgical site infections (SSIs), were also analyzed.

During surgery, under sterile conditions, we collected bile samples by puncturing the common bile duct and gallbladder. Those samples underwent standard microbiological assays for microbes identification. In absence of microbial growth for 48 h, the samples were considered sterile.

Each postoperative complications were defined according to internationally recognized standards [10,11,12,13]; all PDs were performed as previously described [14].

All patients received intravenous antibiotic prophylaxis 30–60 min before the skin incision. Additionally, 2 g Cefazolin and 500 mg Metronidazole were administered to patients who did not receive previous biliary stenting, while 1 g meropenem was the antibiotic of choice for patients with biliary stent. An additional dose of antibiotics was administered three or six hours after the first dose. In case of penicillin allergy, Clindamicine 900 mg and Gentamicine (5 mg/Kg) were administered.

During the postoperative course, patients who developed a clinically relevant infection received antibiotic therapy. In these cases, drugs were selected according to the specific diagnosis and antimicrobial susceptibility test.

Patients who underwent palliative biliary and/or gastric bypass for unresectable malignancies were excluded, including patients with incomplete data regarding biliary cultures and/or their postoperative course.

### Statistical Analysis

Bacteria isolated from intra-operative biliary cultures were analyzed for potential association with each postoperative complication using the ANOVA test to analyze of the variance and heterogeneity of bacterial species. The test compares all possible pairs of means. In this study, Tukey test for multiple comparisons was used to evaluate the potential association and the statistical significance between the prevalence of different bacterial species and each complication. In details, the ANOVA test is used to determine the influence that independent variables have on a dependent variable in a regression study. The ANOVA test makes it possible to compare more than two groups at the same time to determine whether a relationship exists among them. The results of the ANOVA make it possible to analyze multiple groups of data to determine the variability between and within samples. The ANOVA test shows if the results are significant overall, but will not show exactly where differences lie. After an ANOVA detects significant results, the Tukey’s test can be used to find out which specific groups (compared with each other) are different. The test compares all possible pairs of means. The odds ratio was computed to evaluate the association between positive biliary culture and each complication, between Endoscopic Retrograde CholangioPancreatography (ERCP) and each complication, between ERCP and positive biliary culture and between DGE and POPF.

Univariate and multivariate logistic regression analyses were performed to assess the influence of recognized risks factors (e.g., male sex, BMI ≥ 30, Wirsung duct diameter ≤ 3 mm, soft pancreas texture) and polymicrobial bacterobilia on the development of POPF. POPF was used as the dependent variable, while male sex, BMI ≥ 30, Wirsung duct diameter ≤ 3 mm, soft pancreas texture and polymicrobial bacterobilia were used as independent ones.

For statistical analyses, GraphPad Prism version 7.00 for Windows (GraphPad Software, La Jolla, CA, USA) and MedCalc Statistical Software version 19.1 (MedCalc Software bv, Ostend, Belgium, 2019) were used.

## 3. Results

Two hundred forty-four patients were found to be eligible for the analysis in the present study.

One hundred forty-two (58.2%) patients were male and 102 (41.8%) female. The median age was 68 years (range 38–85 years) and the median BMI was 24.24 Kg/m^2^ (range 17.24–38.45 Kg/m^2^). One hundred twenty-two patients (50%) had an American Society of Anesthesiologists (ASA) score of I-II and 122 (50%) ASA III-IV.

Preoperative biliary drainage was performed in 146 patients (59.8%); more in detail, 133 (91.1%) underwent ERCP stenting and 13 (8.9%) percutaneous biliary drainage. Neoadjuvant treatment was performed in 26 patients (10.7%): 5 (19.2%) received chemotherapy alone, while 21 (80.8%) underwent radio-chemotherapy. Table 1 shows patient characteristics.

The diameter of the Wirsung duct was <3 mm in 63 patients (25.8%) and ≥3 mm in 181 patients (74.2%), respectively. One hundred sixty-five patients (67.6%) had a soft gland while 79 (32.4%) had a firm one.

The median intraoperative blood loss was 300 mL (range 100–1450 mL) and the median operative time was 375 min (range 135–720 min).

Intraoperative biliary cultures were positive in 162/244 (66.4%) and negative in 82/244 (33.6%). Among patients with bacterobilia, 138 (85.2%) received preoperative biliary drainage. Moreover, preoperative biliary drainage was found to be strongly associated with positive biliary culture (OR = 56 (24–131); *p* < 0.01) and significantly protective against biliary fistula development (OR = 0.43 (0.20–0.9) *p* < 0.05).

Intraoperative data are listed in Table 2.

The proportion of positive biliary culture in the study population was statistically significant (*p* < 0.01).

**Figure 2:**
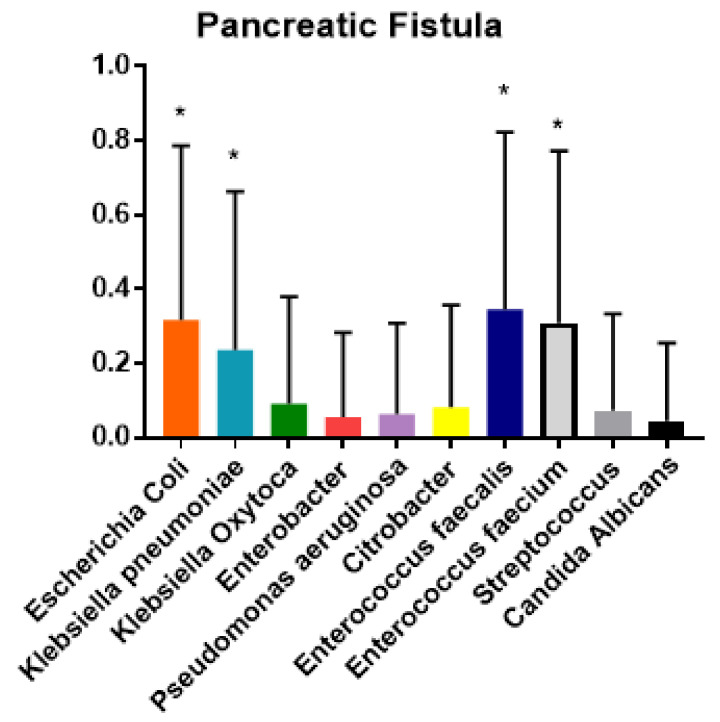
Tukey test for analysis of variance of prevalence (y axis) and bacterial specie (x axis) isolated from biliary culture among pancreatic fistula (POPF). * *p* < 0.01.

**Figure 3:**
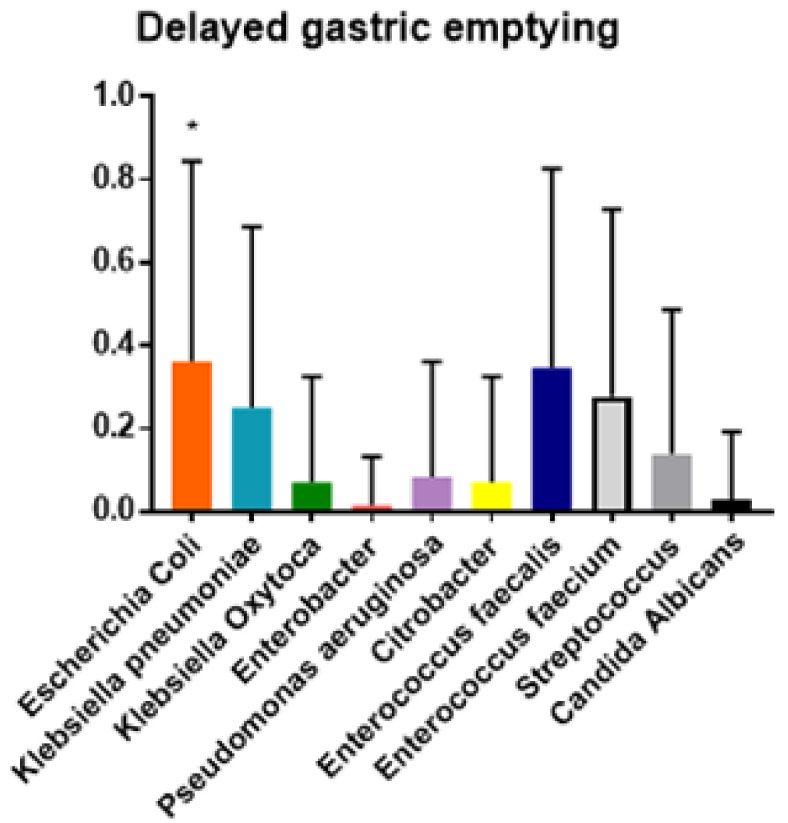
Tukey test for analysis of variance of prevalence (y axis) and bacterial specie (x axis) isolated from biliary culture among delayed gastric emptying (DGE). * *p* < 0.01.

**Figure 4:**
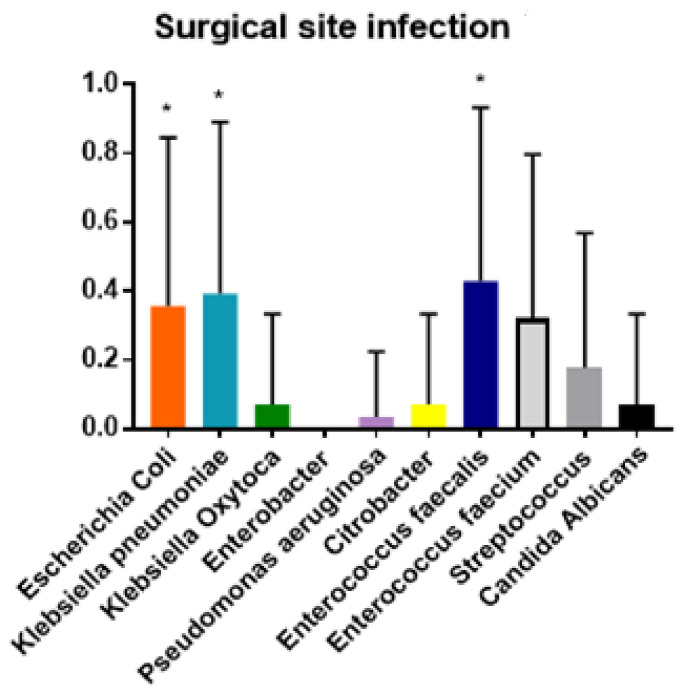
Tukey test for analysis of variance of prevalence (y axis) and bacterial specie (x axis) isolated from biliary culture among surgical site infection (SSI). * *p* < 0.01.

**Figure 5:**
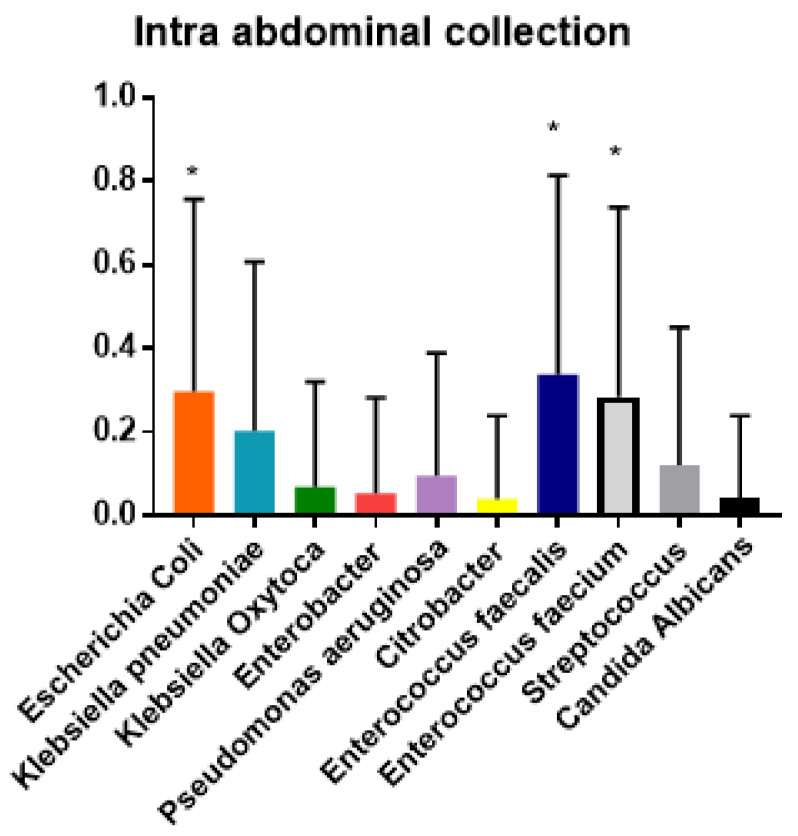
Tukey test for analysis of variance of prevalence (y axis) and bacterial specie (x axis) isolated from biliary culture among intra-abdominal collection. * *p* < 0.01.

The ANOVA analysis demonstrated the prevalence of polymicrobial biliary cultures in postoperative complications. Moreover, the Tukey test demonstrated that the null hypothesis was confirmed and that each bacterial species was more prevalent in some complications (*p* < 0.01) (Figure 2, Figure 3, Figure 4 and Figure 5).

In detail, the Tukey test showed significant prevalence of polymicrobial biliary cultures with *Escherichia coli*, *Klebsiella pneumoniæ* and *Enterococcus fæcalis* in SSIs (*p* < 0.01). Polymicrobial biliary culture positive for *Escherichia coli*, *Klebsiella pneumoniæ*, *Enterococcus fæcalis* and *Enterococcus fæcium* were prevalent in cases of POPF (*p* < 0.01) (Table 3).

DGE was detected in 74 (30.3%) patients. Patients who presented POPF were twice as likely to develop DGE (42 cases; 56.7%) (OR = 1.88 (1.18–3); *p* < 0.01).

Patients with biliary culture positive for *Escherichia coli*, *Enterococcus fæcalis* and *Enterococcus fæcium* showed a higher prevalence of intra-abdominal collection and DGE (*p* < 0.001).

Notably, the presence of only *Escherichia coli* in the biliary culture was significantly associated with DGE when it was registered as a unique complication, in comparison to patients without any complication (OR = 2.94 (1.30–6.70); *p* < 0.01).

As shown in Table 4, in the univariate analysis, only Wirsung duct diameter ≤ 3 mm and soft pancreas were significantly associated with POPF (*p* < 0.0001 and *p* 0.002, respectively).

Patients with Wirsung duct diameter ≤ 3 mm (OR = 8.0115 (CI 1.8292–35.0981); *p* 0.0058) and those with a soft pancreas (OR = 5.0897 (CI 2.2225–11.6559); *p* 0.0001) showed higher risk of developing POPF (Table 5).

## 4. Discussion

Bacterobilia plays a recognized role in determining infectious complications after pancreatic surgery, and preoperative biliary drainage has been widely documented as a main cause of biliary colonization.

Although the potential negative consequences of preoperative biliary drainage have been demonstrated, and its use recommended only for patients who cannot undergo early surgery, in daily clinical practice, this indication is often overlooked.

Jaundiced patients with periampullary neoplasms still receive biliary drainage in the absence of cholangitis before early tumor removal has been fully excluded [15].

Moreover, despite the fact that it has been reported that bacterobilia is associated with even noninfectious complications, such as POPF [6], its relationship with other common complications after PD, such as DGE, has not been well defined.

Nevertheless, to the best of our knowledge, there is a lack of data regarding the study of prevalence of different biliary bacterial species in postoperative complications after PD.

Our findings showed that intraoperative biliary culture was positive in a significant proportion of patients undergoing pancreatic surgery. The high rate of preoperative biliary drainage, significantly associated with the presence biliary culture, justify the significant proportion of bacterobilia in our series. The non-negligible rate of preoperative biliary drainage we reported was mainly due to the inclusion of many PDAC patients who had already received urgent biliary stenting in other hospitals.

Despite this, according to the results of this study, preoperative biliary stenting was shown to be significantly protective against postoperative biliary fistula. This finding must be interpreted with caution. According to Andrianello [16], who demonstrated in a series of more than 1600 PDs how the diameter of the bile duct is the main independent factor to predict biliary fistula after PD, it is our opinion that more than the ERCP itself, bile duct dilatation contributed to the lower rate of biliary anastomotic leakage detected in these patients.

As reported by Krüger [17], our data confirm that preoperative biliary drainage increases the risk of bile colonization. Indeed, in accordance with others [15], it is also our opinion that drainage should be carried out only in the presence of cholangitis or whenever early upfront surgery cannot be performed.

Furthermore, a strategy to reduce the rate of positive biliary cultures following endoscopic preoperative biliary drainage, and consequently the postoperative complications associated with positive biliary cultures, could be to encourage hospital boards to perform microbiological surveillance of duodenoscopes. In order to prevent bacterial transmission and, consequently, patient colonization, Ciccozzi suggested that duodenoscope surveillance should be routinely performed after the reprocessing procedure of all instruments used for ERCP [18].

Analysis of variance and a Tukey test showed that specific prevalence of biliary bacterial species are present in patients with complications after PD.

In the majority of the cases, biliary bacterial flora is polymicrobial. Notably, polymicrobial biliary cultures were significantly associated not only with SSIs, but also with POPF and intra-abdominal collections.

These results are in agreement with those of other authors who reported on how polymicrobial biliary flora is predictive of polymicrobial overgrowth of bacteria in intra-abdominal collections and wound infections after pancreatic surgery [19,20].

Regarding POPF, our data could suggest a relationship between polymicrobial Gram-negative bacterobilia (*Escherichia coli*, *Klebsiella pneumoniæ*, *Enterococcus fæcalis* and *Enterococcus fæcium*) and this feared complication.

Other authors have already investigated the relationship between bacterobilia and POPF and presented different theories to clarify this association.

In a retrospective review of 264 PDs, Ohgi showed that the incidence of clinically relevant postoperative pancreatic fistulas (CR-POPF) was significantly higher in patients with positive biliary culture collected during the surgery. Moreover, the microorganisms detected in the surgical drain fluid of these patients were the same as those isolated from the biliary culture. Since positive biliary culture was not found to be associated with biochemical leaks, the authors speculated a possible role of bacteria in the development of CR-POPF via colonization of the ascitic fluid [8].

In our research, the analysis of each bacterial species isolated in patients with postoperative complications demonstrated a prevalence of Enterococci in POPF. This result partly disagrees with a recent report by Heckler, who, in a retrospective study on 289 PDs performed on malignancies after preoperative biliary drainage, demonstrated that *Escherichia coli* bacterobilia was associated with severe POPF, while Enterococci, although representative of the most isolated microbes in the bile duct swab, was mainly associated with lymphatic fistula [8].

In 2015, Shogan suggested that the impairment of collagen repair caused by Enteroccocci could be responsible for anastomotic failure in colorectal surgery [21]. In 2018, Belmouhand [22] demonstrated, for the first time, how the presence of Enterococci, isolated from cultures in surgical drains in different postoperative days after PD, was significantly associated with POPF. According to Shogan, this author also suggested a role of the proteases produced by these germs that degrade the collagen of the extracellular matrix of pancreatic anastomoses.

As with Belmouhand’s study, ours also has limitations, such as the retrospective design and the absence of biochemical analyses to confirm the negative effect of bacterial proteases. However, our microbiological analysis was conducted on intraoperative bile sampling, and not on fluid collected in the abdominal drains over the course of several postoperative days, increasing the risk of accidental contamination. In addition, in Belmouhand’s research, patients also routinely received antibiotic therapy with cefuroxime and metronidazole for the first three days after surgery.

Our results may support the hypothesis of Loos, who recently assessed the impact of microbiologic pathogens isolated from POPF juice and abdominal swabs in cases of relaparotomy after pancreatic surgery. In Loos’ research, the postoperative presence of Enterococci in abdominal fluids was significantly associated with worse outcome of POPF. According to Loos, contamination of the abdominal cavity during the reconstructive phase of PD, in association with anastomotic leakage, may explain the presence of bacteria isolated in POPF [23].

Regarding DGE, our experience confirmed that it is usually associated with POPF, and we observed a significant higher prevalence of polymicrobial Gram-negative bacterobilia (*Escherichia coli*, *Enterococcus fæcalis* and *Enterococcus fæcium*). These results are in line with those reported in Chen’s meta-analysis. Chen reported an increased rate of DGE in patients affected by POPF, and showed how the presence of bilicultures was significantly associated with DGE [5]. In agreement with others [24], Chen attributed DGE to the effect of local inflammation caused by pancreatic juices, more than to an altered biliary microbiome.

Although DGE is often the consequence of other complications, primary DGE has been reported in 12% of patients who underwent PD; the underlying causes for this remain poorly understood [25].

Notably, in our research, an interesting result emerged from the significantly higher prevalence of monomicrobial *Escherichia coli* bacterobilia and DGE when this developed as a singular complication. Eisenberg already reported the possible association between bacteria and DGE [25]; this author found that abdominal infections were a significant prognostic factor for primary DGE. However, Eisenberg noted that it cannot be determined whether DGE was really a consequence of an associated abdominal infection or developed independently. If further confirmed, our results may support the Eisenberg theory, i.e., that subclinical abdominal infections may be present in cases of primary DGE. Nevertheless, it has to be considered that under normal conditions, *Escherichia coli* is absent in the microbial flora colonizing the gastric mucosa [26], and that, as previously demonstrated in rats, *Escherichia coli* lipopolysaccharide is able to induce a significant delay of the gastric emptying [27]. Moreover, different studies have demonstrated that the peptidoglycans of microbiota activate an inflammatory response with a consequent release of cytokines that results in dysfunction of intestinal smooth muscle and delayed gastrointestinal motility [28].

Therefore, antibiotic therapy tailored to the antimicrobial susceptibility test performed on biliary samples collected intraoperatively could be applied in these cases.

Furthermore, since the gut microbiota is the “conductor in the orchestra” of immune-neuro-endocrine communication [29], interacting with the enteric and central nervous systems along the “gut-brain-axis”, gut dysbiosis could play an important role in delayed gastro-intestinal motility. Bacteria such as *Escherichia coli* can produce and/or consume neurotransmitters, including gamma-aminobutyric acid (GABA) which is the main inhibitory neurotransmitter in intestinal motility and gastric emptying. It is known that altered GABAergic neurotransmission can induce disruption of important functions of the enteric nervous system, such as gastric emptying [30,31].

Since gut motility is a complex balance involving immune and nervous system function, and considering the beneficial effect of probiotics demonstrated by Dimidi [32], if our results are confirmed in larger prospective studies, we can also hypothesize that the modulation of microbiota through the use of probiotics could be helpful in the management of DGE in pancreatic surgery.

This study presents several limitations, including its retrospective design and the fact that patients who had undergone previous neoadjuvant treatments were not excluded. However, according to Goel [33], potential differences in the biliary microbiome detected in patients after neoadjuvant treatment may have been due to the preoperative stenting that these patients usually receive, more than to the neoadjuvant treatment itself.

## 5. Conclusions

Intraoperative biliary culture represents a valid tool for follow-up and prognoses of postoperative complications after PD. Polymicrobial bacterobilia are prevalent in cases of major postoperative complications, such as SSI and POPF, while monomicrobial *Escherichia coli* bacterobilia is prevalent when DGE occurs as a unique complication.

If the prevalences of these microbiomes is confirmed, antibiotics tailored to the results of an antimicrobial susceptibility test performed on intraoperatively collected biliary samples and gut microbiome management may improve outcomes of PD.

## Figures and Tables

**Table 1:** Demographic and clinical characteristics of the series.

Study Population (244 Patients)	
Age, years Median (range)	68 (38–85)
Gender, *n* (%)	
Male	142 (58.2)
Female	102 (41.8)
BMI, Kg/m^2^ Median (range)	24.24 (17.24–38.45)
Neoadjuvant Treatment, *n* (%)	26 (10.7)
CT	5 (19.2)
CT-RT	21 (80.8)
ASA score, *n* (%)	
I–II	122 (50)
III–IV	122 (50)
Preoperative biliary drainage, *n* (%)	146 (59.8)

(BMI = Body Mass Index; CT = chemotherapy alone; CT-RT: radiochemotherapy; ASA = American Society of Anesthesiologists Score).

**Table 2:** Surgical intraoperative characteristics and biliary culture data.

Study Population (244 Patients)	
Wirsung duct diameter, *n* (%)	
<3 mm	63 (25.8)
≥3 mm	181 (74.2)
Pancreatic texture, *n* (%)	
Soft	165 (67.6)
Firm	79 (32.4)
Blood loss median, mL (range)	300 (100–1450)
Operative time median, min (range)	375 (135–720)
Positive Biliary cultures, *n* (%) Previous preoperative biliary drainage, *n* (%)	162 (66.4) 138 (85.2)

**Table 3:** Tukey test showing prevalence of bacterial species isolated from biliary cultures in different postoperative complications.

	*E. coli* *n*, (%)	*K. pneumoniae* *n*, (%)	*E. faecalis* *n*, (%)	*E. faecium* *n*, (%)	*p*
SSI	10 (4.1)	11 (4.5)	12 (4.9)	-	<0.01
POPF	34 (13.9)	25 (10.2)	38 (15.6)	32 (13.1)	<0.01
DGE	27 (11.1)		26 (10.7)	21 (8.6)	<0.01

SSI: surgical site infections; POPF: postoperative pancreatic fistula; DGE: delayed gastric emptying.

**Table 4:** Univariate analysis showing risks factors for POPF.

Risks Factors	Odds Ratio (Confidence Interval)	*p*
Male sex BMI ≥ 30 Wirsung duct diameter ≤ 3 mm Soft pancreas texture Polimicrobial bacterobilia	1.3584 (0.6678–2.7628) 1.0353 (0.3329–3.2197) 5.9935 (2.6765–13.4209) 9.8790 (2.3062–42,3178) 0.7395 (0.3722–1.4690)	0.3978 0.9522 <0.0001 * 0.002 * 0.388

* Statistical significant.

**Table 5:** Logistic regression analysis showing risks factors for POPF.

Statistical Significant Variables Included in the Model	Odds Ratio (Confidence Interval)	*p*
Wirsung duct diameter ≤ 3 mm Soft pancreas texture	8.0115 (18,292–350,891) 5.0897 (22,225–116,559)	0.0058 * 0.0001 *
Other independent variables included in the model that did not reach statistical significance		
BMI ≥ 30 Male sex Polimicrobial Bacterobilia		

* Statistical significant.

## Data Availability

The data presented in this study are available on request from the corresponding author.
